# Increased adipose tissue is associated with improved overall survival, independent of skeletal muscle mass in non‐small cell lung cancer

**DOI:** 10.1002/jcsm.13333

**Published:** 2023-09-19

**Authors:** Junli Tao, Jiayang Fang, Lihua Chen, Changyu Liang, Bohui Chen, Zhenyu Wang, Yongzhong Wu, Jiuquan Zhang

**Affiliations:** ^1^ Department of Radiology Chongqing University Cancer Hospital & Chongqing Cancer Institute & Chongqing Cancer Hospital Chongqing P.R. China; ^2^ Key Laboratory for Biorheological Science and Technology of Ministry of Education (Chongqing University) Chongqing University Cancer Hospital & Chongqing Cancer Institute & Chongqing Cancer Hospital Chongqing P.R. China; ^3^ Department of radiotherapy Chongqing University Cancer Hospital & Chongqing Cancer Institute & Chongqing Cancer Hospital Chongqing P.R. China

**Keywords:** Adipose tissue, Body composition, Body mass index, Non‐small cell lung cancer, Skeletal muscle

## Abstract

**Background:**

The prognostic significance of non‐cancer‐related prognostic factors, such as body composition, has gained extensive attention in oncological research. Compared with sarcopenia, the prognostic significance of adipose tissue for overall survival in non‐small cell lung cancer remains unclear. We investigated the prognostic value of skeletal muscle and adipose tissue in patients with non‐small cell lung cancer.

**Methods:**

This retrospective study included 4434 patients diagnosed with non‐small cell lung cancer between January 2014 and December 2016. Cross‐sectional areas of skeletal muscle and subcutaneous fat were measured, and the pericardial fat volume was automatically calculated. The skeletal muscle index and subcutaneous fat index were calculated as skeletal muscle area and subcutaneous fat area divided by height squared, respectively, and the pericardial fat index was calculated as pericardial fat volume divided by body surface area. The association between body composition and outcomes was evaluated using Cox proportional hazards model.

**Results:**

A total of 750 patients (501 males [66.8%] and 249 females [33.2%]; mean age, 60.9 ± 9.8 years) were included. Sarcopenia (60.8% vs. 52.7%; *P* < 0.001), decreased subcutaneous fat index (51.4% vs. 25.2%; *P* < 0.001) and decreased pericardial fat index (55.4% vs. 16.5%; *P* < 0.001) were more commonly found in the deceased group than survived group. In multivariable Cox regression analysis, after adjusting for clinical variables, increased subcutaneous fat index (hazard ratio [HR] = 0.56, 95% confidence interval [CI]: 0.47–0.66, *P* < 0.001) and increased pericardial fat index (HR = 0.47, 95% CI: 0.40–0.56, *P* < 0.001) were associated with longer overall survival. For stage I–III patients, increased subcutaneous fat index (HR = 0.62, 95% CI: 0.48–0.76, *P* < 0.001) and increased pericardial fat index (HR = 0.43, 95% CI: 0.34–0.54, *P* < 0.001) were associated with better 5‐year overall survival rate. Similar results were recorded in stage IV patients. For patients with surgery, the prognostic value of increased subcutaneous fat index (HR = 0.60, 95% CI: 0.44–0.80, *P* = 0.001) and increased pericardial fat index (HR = 0.51, 95% CI: 0.38–0.68, *P* < 0.001) remained and predicted favourable overall survival. Non‐surgical patients showed similar results as surgical patients. No association was noted between sarcopenia and overall survival (*P* > 0.05).

**Conclusions:**

Increased subcutaneous fat index and pericardial fat index were associated with a higher 5‐year overall survival rate, independent of sarcopenia, in non‐small cell lung cancer and may indicate a reduced risk of non‐cancer‐related death.

## Introduction

Lung cancer is the most common diagnosed cancer in men and the second in women, which leads to cancer‐related deaths in both sexes.^1^ Non‐small cell lung cancer (NSCLC), mainly squamous cell carcinoma and adenocarcinoma, accounts for 80–85% of all lung cancer cases.^2^ Despite considerable advances in treatment strategies, the prognosis of NSCLC patients remains poor, with a 5‐year overall survival (OS) rate of 15%.^3^ Currently, the gold standard for prognostic prediction in NSCLC patients is tumour‐node‐metastasis (TNM) staging.^4^ However, the clinical outcomes can differ among NSCLC patients with the same TNM staging.^5^ Therefore, it is necessary to investigate other prognostic factors to improve the survival rate of NSCLC patients.

Previous studies have shown that the short‐ and long‐term outcomes of lung cancer also depend on host factors.^6^, ^7^ Recently, the prognostic significance of body composition has gained considerable attention in oncological research.^8^ Body composition primarily consists of skeletal muscle and adipose tissue (including subcutaneous fat and visceral fat), whereas body mass index (BMI) comprises both. Studies have found that BMI affects the survival of NSCLC patients, with underweight patients having a lower survival rate.^9^ However, some patients with the same BMI have different prognosis,^10^ which may be related to the different contributions of skeletal muscle and fat to the patient's prognosis. Unfortunately, BMI cannot distinguish between skeletal muscle and fat or independently assess their impact on the prognosis of NSCLC patients.^11^

Computed tomography (CT) is considered the gold standard for assessing body composition, allowing non‐invasive and objective simultaneous quantification of the amount of skeletal muscle and adipose tissue at the desired anatomical level.^12^ Chest CT is routinely performed in NSCLC patients during the initial diagnosis and follow‐up assessment and may be the best option for the regular assessment of thoracic skeletal muscle and fat.^13^ Previous studies have shown that sarcopenia, which is indicated by reduced pectoralis muscle mass on CT, is significantly associated with poor OS in NSCLC.^14^ However, the relationship between OS and fat in NSCLC patients has been poorly reported. Previous studies have found that pericardial fat, a representative of visceral fat, is associated with NSCLC prognosis,^15^ and low pericardial fat volume may be associated with poor OS.^16^ To date, the effects of skeletal muscle and fat on the prognosis of NSCLC patients remain unknown.

In this study, we aimed to evaluate the association between baseline adipose tissue (including subcutaneous fat and pericardial fat) and 5‐year OS in NSCLC patients. The prognostic value of adipose tissue was explored to determine its association with skeletal muscle.

## Methods

This retrospective analysis was approved by the institutional review board of our hospital, which waived the requirement for written informed consent.

### Patients

**Figure 1:**
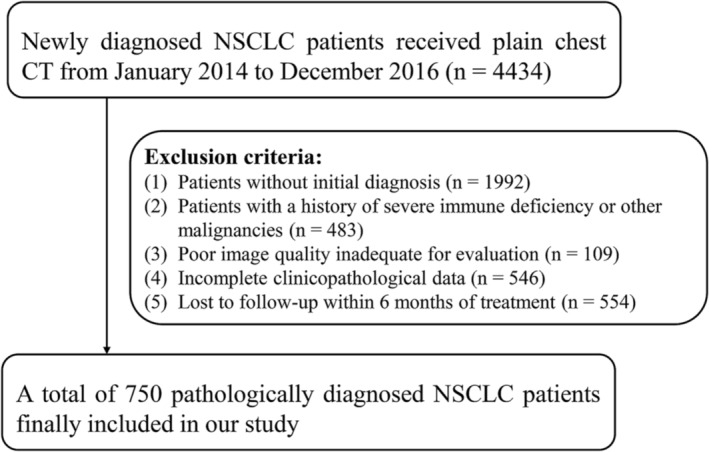
Flowchart of patient inclusion and exclusion criteria.

We retrospectively reviewed the electronic medical records of 4434 patients diagnosed with NSCLC at a single tertiary hospital between January 2014 and December 2016. The inclusion criteria were as follows: (1) initial diagnosis, (2) chest CT thin‐slice images (slice thickness = 1 mm) with a mediastinal window available on our Picture Archiving and Communication System and (3) complete clinicopathological data. The exclusion criteria were as follows: (1) history of severe immune deficiency or other malignancies, (2) poor image quality that was inadequate for evaluation and (3) loss to follow‐up within 6 months of treatment. The flowchart of patients is shown in *Figure* 1.

### Data collection and follow‐up

Patient demographics (age, sex, height, weight and BMI), smoking history, family history, diabetes mellitus, hypertension, coronary artery calcification, carcinoembryonic antigen (CEA), emphysema, histologic subtype, TNM staging according to the 8th edition and types of initial treatment (surgery, radiotherapy, chemotherapy, targeted therapy and immune therapy) were recorded. BMI was calculated as weight divided by height squared (kg/m^2^). Because all our patients were Asians, underweight was defined as BMI < 18.5 kg/m^2^, normal as BMI 18.5–22.9 kg/m^2^, overweight as BMI 23.0–24.9 kg/m^2^ and obese as BMI ≥ 25 kg/m^2^.^11^ The primary outcome was the 5‐year OS rate, with OS duration defined as the time from the date of surgery or the first cycle of chemotherapy/radiotherapy to death from any cause or the last follow‐up.

### Computed tomography scans

Chest CT was conducted using the 64‐multidetector Philips Brilliance CT with standard thoracic scanning protocols. The acquisition parameters were as following: tube current, 80–160 mAs; tube voltage, 120–130 kV; slice thickness, 0.7–1 mm; slice gap, 0.5–0.7 mm; matrix, 512 × 512; and convolution kernel, B40, standard (B).

### Computed tomography quantifies body composition

The quantitative analysis was performed on Simens syngo via VB20.

**Figure 2:**
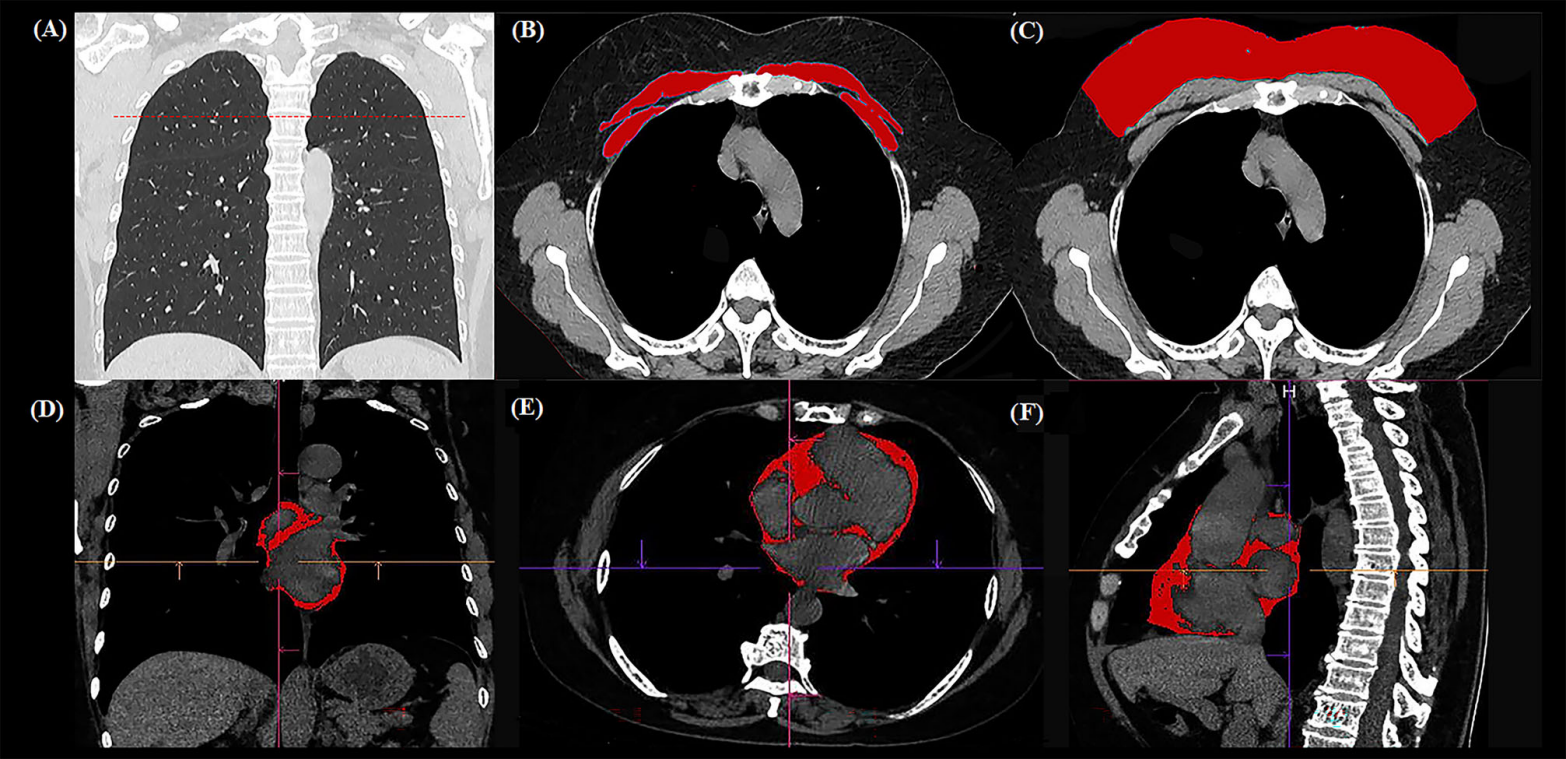
Schematic diagram of body composition evaluation. Body composition was measured at the level of the fourth thoracic vertebra transverse process on CT (A). The pectoralis major and minor muscle border was outlined, and the pectoralis area was quantified according to the Hounsfield unit threshold (−29 to 150) (B). The subcutaneous fat area was measured on the same image in front of the pectoralis muscles with margins extending to the pectoralis major, Hounsfield unit threshold of subcutaneous fat was −190 to −30 (C). Pericardial fat extends vertically from the right pulmonary artery to the diaphragm and horizontally from the left edge of the apex to the right edge of the atrium, CT densities between −190 and −30 HU the pericardial fat volume was automatically calculated (D–F).

The images of staging CT scan were used for the analysis. At the level of the fourth thoracic vertebra (Th4) on non‐contrast chest CT images, the bilateral pectoralis major and pectoralis minor muscles were manually delineated at the utmost border by a radiologist (F. J. Y., reader 1) with 7‐year experience in chest imaging (blinded to patient's information); then, the skeletal muscle area (cm^2^) was calculated (*Figure* 2A,B). The subcutaneous fat area (cm^2^) was measured on the same image in front of the pectoralis muscles, with the margins extending to the pectoralis major (*Figure* 2C). The Hounsfield unit threshold of skeletal muscle was −29 to 150 and that of subcutaneous fat was −190 to −30.^17^, ^18^ Pericardial fat extends vertically from the right pulmonary artery to the diaphragm and horizontally from the left edge of the apex to the right edge of the atrium, and the area of fat was identified by CT densities between −190 and −30 HU. Pericardial fat volume (cm^3^) was automatically calculated once the pericardial fat was identified^16^ (*Figure* 2D–F).

The skeletal muscle and subcutaneous fat index (SMI and SFI, cm^2^/m^2^) were calculated as skeletal muscle area and subcutaneous fat area divided by height squared (m^2^), respectively, and the pericardial fat index (PFI, cm^3^/m^2^) was calculated as pericardial fat volume divided by body surface area. Body surface area was calculated using Stevenson's formula.^19^

To test the reliability of skeletal muscle area, subcutaneous fat area, and pericardial fat volume determined using CT, 1 month after the quantification, reader 1 and another radiologist (T.J.L, reader 2) with 8‐year experience in thoracic imaging randomly selected 150 patients and independently performed the delineation and measurement. The intra‐ and interobserver correlation coefficient (ICC) values were generated. ICC > 0.8 indicated good agreement.

### Statistical analysis

Statistical calculations and corresponding illustrations were performed using GraphPad Prism 8 (GraphPad Software, Inc., USA) and SPSS Statistics (version 27.0; IBM Corp., USA). Two‐sided *P*‐values < 0.05 indicated statistically significant differences.

Categorical variables are expressed as frequency and percentage. The Kolmogorov–Smirnov test was used to examine the distribution of continuous variables. All continuous variables were abnormally distributed and are described as median and interquartile range. The chi‐square test was used to compare categorical data between the two groups, and the Mann–Whitney *U* test was used for continuous data.

The cut‐off values for SMI, SFI and PFI were generated using X‐tile software (Yale University, USA) according to sex, because the standard cut‐off values for SMI, SFI and PFI are not currently available at the Th4 level. This process is similar to Lang et al. described.^20^ First, we chose a specific cut‐off value that partitioned our patients into one group above and another group below the cut‐off value. Then, Kaplan–Meier curves from both groups were generated, and the log‐rank test was used to evaluate significant differences between the emanative survival curves. Finally, the optimal cut‐off value was defined based on the ‘minimal *P*‐value approach’, which was the value that produced Kaplan–Meier curves with the lowest *P*‐value in the log‐rank test.

For OS analysis, Kaplan–Meier curves were generated and compared using the log‐rank test. Univariate and multivariate Cox regression analyses were performed using a Cox proportional hazards model. Variables with *P* < 0.1 in the univariate analysis were included in the multivariate analysis. Hazard ratios (HRs) and the corresponding 95% confidence intervals (CIs) were calculated.

## Results

### Baseline clinicopathological characteristics

A total of 750 NSCLC patients [501 males (66.8%) and 249 females (33.2%); mean age, 60.9 ± 9.8 years] were included in our study. The clinicopathological characteristics of the patients are presented in *Table* 1. Most patients had no family history (*n* = 686, 91.5%), coronary calcification (*n* = 476, 63.5%), diabetes mellitus (*n* = 664, 88.5%) or hypertension (*n* = 595, 79.3%). Nearly half of the patients were never smokers (*n* = 340, 45.3%), had normal weight (*n* = 338, 45.1%), stage IV disease (*n* = 353, 47.0%), emphysema (*n* = 335, 44.7%) and increased CEA level (*n* = 324, 43.2%). More than half of the patients had adenocarcinoma (*n* = 439, 58.5%) or metastasis (*n* = 397, 52.9%). Furthermore, 291 patients (38.8%) were in T2 staging, and 226 patients (30.2%) were in N3 staging. A total of 257 (34.3%) patients were initially treated with surgery; the initial treatments in the remaining patients included radiotherapy (*n* = 144, 19.2%), chemotherapy (*n* = 211, 28.1%), targeted therapy (*n* = 98, 13.1%) and immunotherapy (*n* = 40, 5.3%).

**Table 1:** Clinicopathological characteristics of patients

Characteristic	Patients (*n* = 750)
Age (year)^a^	62 (53–68)
Gender	
Male	501 (66.8)
Female	249 (33.2)
Smoking history	
Current	280 (37.3)
Never	340 (45.3)
Former	130 (17.4)
Family history	
Yes	64 (8.5)
No	686 (91.5)
CEA (ng/mL)	
Increased (>5)	324 (43.2)
Normal (≤5)	426 (56.8)
BMI (kg/m^2^)^a^	22.5 ± 3.3
BMI (kg/m^2^) category	
Underweight (<18.5)	89 (11.9)
Normal (18.5–22.9)	341 (45.5)
Overweight (23.0–24.9)	164 (21.9)
Obese (≥25)	156 (20.8)
Histologic type	
Squamous cell carcinoma	265 (35.3)
Adenocarcinoma	439 (58.5)
Other NSCLC	46 (6.1)
Coronary calcification	
Yes	274 (36.5)
No	476 (63.5)
Diabetes mellitus	
Yes	86 (11.5)
No	664 (88.5)
Hypertension	
Yes	155 (20.7)
No	595 (79.3)
Emphysema	
Yes	335 (44.7)
No	415 (55.3)
Stage	
1	116 (15.5)
2	78 (10.4)
3	203 (27.1)
4	353 (47.1)
Initial treatment	
Surgery	257 (34.3)
Radiotherapy	144 (19.2)
Chemotherapy	211 (28.1)
Targeted therapy	98 (13.1)
Immune therapy	40 (5.3)

TNM staging was based on the 8th AJCC system for lung cancer.

BMI, body mass index.

^a^ Numbers are medians with interquartile ranges in parentheses and the rest data are numbers of patients with percentages in parentheses.

### Comparison of patients' clinicopathological characteristics and body composition based on mortality

Significant differences were found in CEA level (increased, 30.7% vs. 45.7%; *P* = 0.002), BMI (23.0 kg/m^2^ vs. 22.4 kg/m^2^; *P* = 0.001), coronary calcification (yes, 23.6% vs. 39.2%; *P* = 0.001), TNM staging (T1, 12.6% vs. 16.9%, *P* = 0.039; N0, 40.9% vs. 27.9%, *P* = 0.009; M0, 70.1% vs. 49.4%, *P* < 0.001), clinical stage (I, 25.2% vs. 13.5%; *P* < 0.001) and initial treatment (surgery, 74.0% vs. 26.2%, *P* < 0.001) between the survived and deceased patients. However, no statistical differences were found in the other clinicopathological variables between the survived and deceased groups (*Table* 2).

**Table 2:** Comparison of patients' clinicopathological characteristics and body composition on the basis of mortality

Characteristic	Survived (*n* = 127)	Died (*n* = 623)	*P* value
Age (year)^a^	60.1 (53–66)	61.1 (53–68)	0.02
Gender			0.14
Male	92 (72.4)	409 (65.7)	
Female	35 (27.6)	214 (34.3)	
Smoking history			0.41
Current	54 (42.5)	226 (36.3)	
Never	52 (40.9)	288 (46.2)	
Former	21 (16.5)	109 (17.5)	
Family history			0.52
Yes	9 (7.1)	55 (8.8)	
No	118 (92.9)	568 (91.2)	
CEA (ng/mL)			0.002
Increased	39 (30.7)	285 (45.7)	
Normal	88 (69.3)	338 (54.3)	
BMI (kg/m^2^)^a^	23.0 (21.4–25.8)	22.4 (19.8–24.3)	0.001
BMI (kg/m^2^) category			0.002
Underweight (<18.5)	8 (6.3)	81 (13.0)	
Normal (18.5–22.9)	54 (41.7)	287 (45.7)	
Overweight (23.0–24.9)	24 (20.5)	140 (23.8)	
Obese (≥25)	41 (31.5)	115 (17.5)	
Histologic type			0.69
Squamous cell carcinoma	41 (32.3)	224 (36.0)	
Adenocarcinoma	77 (60.6)	362 (58.1)	
Other NSCLC	9 (7.1)	37 (5.9)	
Coronary calcification			0.001
Yes	30 (23.6)	244 (39.2)	
No	97 (76.4)	379 (60.8)	
Diabetes mellitus			0.1
Yes	20 (15.7)	66 (10.6)	
No	107 (84.3)	557 (89.4)	
Hypertension			0.31
Yes	22 (17.3)	133 (21.3)	
No	105 (82.7)	490 (78.7)	
Emphysema			0.09
Yes	48 (37.8)	287 (46.1)	
No	79 (62.2)	336 (53.9)	
T			0.04
1	16 (12.6)	105 (16.9)	
2	63 (49.6)	228 (36.6)	
3	22 (17.3)	112 (18.0)	
4	26 (20.5)	178 (28.6)	
N			0.01
0	52 (40.9)	174 (27.9)	
1	17 (13.4)	80 (12.8)	
2	33 (26.0)	168 (27.0)	
3	25 (19.7)	201 (32.3)	
M			<0.001
0	89 (70.1)	308 (49.4)	
1	38 (29.9)	315 (50.6)	
Clinical stage			<0.001
1	32 (25.2)	84 (13.5)	
2	19 (15.0)	59 (9.5)	
3	38 (29.9)	165 (26.5)	
4	38 (29.9)	315 (50.6)	
Initial treatment			<0.001
Surgery	94 (74.0)	163 (26.2)	
Non‐surgery	33 (26.0)	460 (73.8)	
SMI (cm^2^/m^2^)^a^			
Male	20.2 (18.2–22.8)	18.3 (14.5–21.7)	<0.001
Female	21.2 (15.4–28.8)	15.2 (12.4–19.3)	0.001
SFI (cm^2^/m^2^)^a^			
Male	34.6 (17.8–43.1)	18.4 (12.1–26.1)	<0.001
Female	47.7 (34.4–58.3)	27.8 (21.1–37.8)	<0.001
PFI (cm^3^/m^2^)^a^			
Male	99.1 (84.5–120.1)	72.5 (51.5–104.4)	<0.001
Female	111.3 (90.1–128)	87.2 (71.9–114.4)	0.001

TNM staging was based on the 8th AJCC system for lung cancer.

BMI, body mass index; PFI, pericardial fat index; SFI, subcutaneous fat index.

^a^ Numbers are medians with interquartile ranges in parentheses and the rest data are numbers of patients with percentages in parentheses.

**Figure 3:**
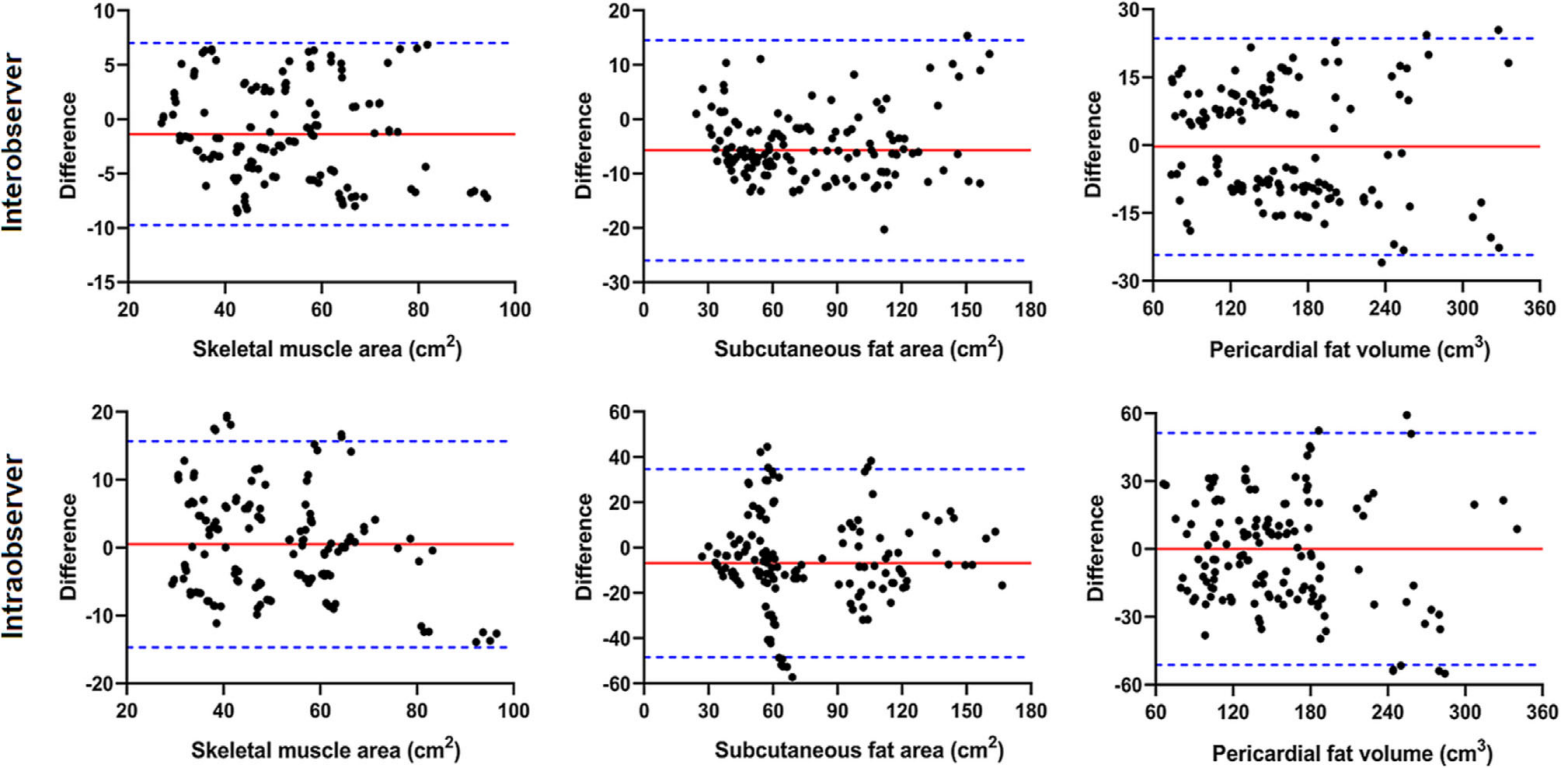
Bland–Altman plots show differences between inter‐observer and intra‐observer measurements after 1 month. The upper and lower dashed horizontal lines represent the upper and lower limits of the 95% confidence interval, and the solid middle line represents the average of the differences.

The ICC values for interobserver reliability were as follows: skeletal muscle area: 0.95, subcutaneous fat area: 0.95 and pericardial fat volume: 0.97; the ICC values for intra‐observer reliability were as follows: skeletal muscle area: 0.88, subcutaneous fat area: 0.81 and pericardial fat volume: 0.90. Bland–Altman analysis showed that the three parameters had good inter‐ and intra‐observer reliability (*Figure* 3). Therefore, the body composition values measured by reader 1 were used for subsequent analysis. The cut‐off values of SMI, SFI and PFI generated according to sex were 14.7 cm^2^/m^2^, 28.9 cm^2^/m^2^ and 85.3 cm^3^/m^2^, respectively, for females and 18.1 cm^2^/m^2^, 21.0 cm^2^/m^2^ and 81 cm^3^/m^2^ for males, respectively. SMI, SFI and PFI less than or equal to the cut‐off values were indicative of sarcopenia, decreased SFI and decreased PFI, respectively. SFI and PFI greater than the cut‐off values were defined as increased SFI and PFI.

SMI (18.3 cm^2^/m^2^ vs. 20.2 cm^2^/m^2^ for males and 15.2 cm^2^/m^2^ vs. 21.2 cm^2^/m^2^ for females; *P* < 0.001 and *P* = 0.001, respectively), SFI (18.4 cm^2^/m^2^ vs. 34.6 cm^2^/m^2^ for males and 27.8 cm^2^/m^2^ vs. 47.7 cm^2^/m^2^ for females; both *P* < 0.001) and PFI (72.5 cm^3^/m^2^ vs. 99.1 cm^3^/m^2^ for males and 87.2 cm^3^/m^2^ vs. 111.3 cm^3^/m^2^ for females; *P* < 0.001 and *P* = 0.001, respectively) were significantly decreased in deceased patients compared with that in survived patients.

### Body composition and 5‐year overall survival rate

**Figure 4:**
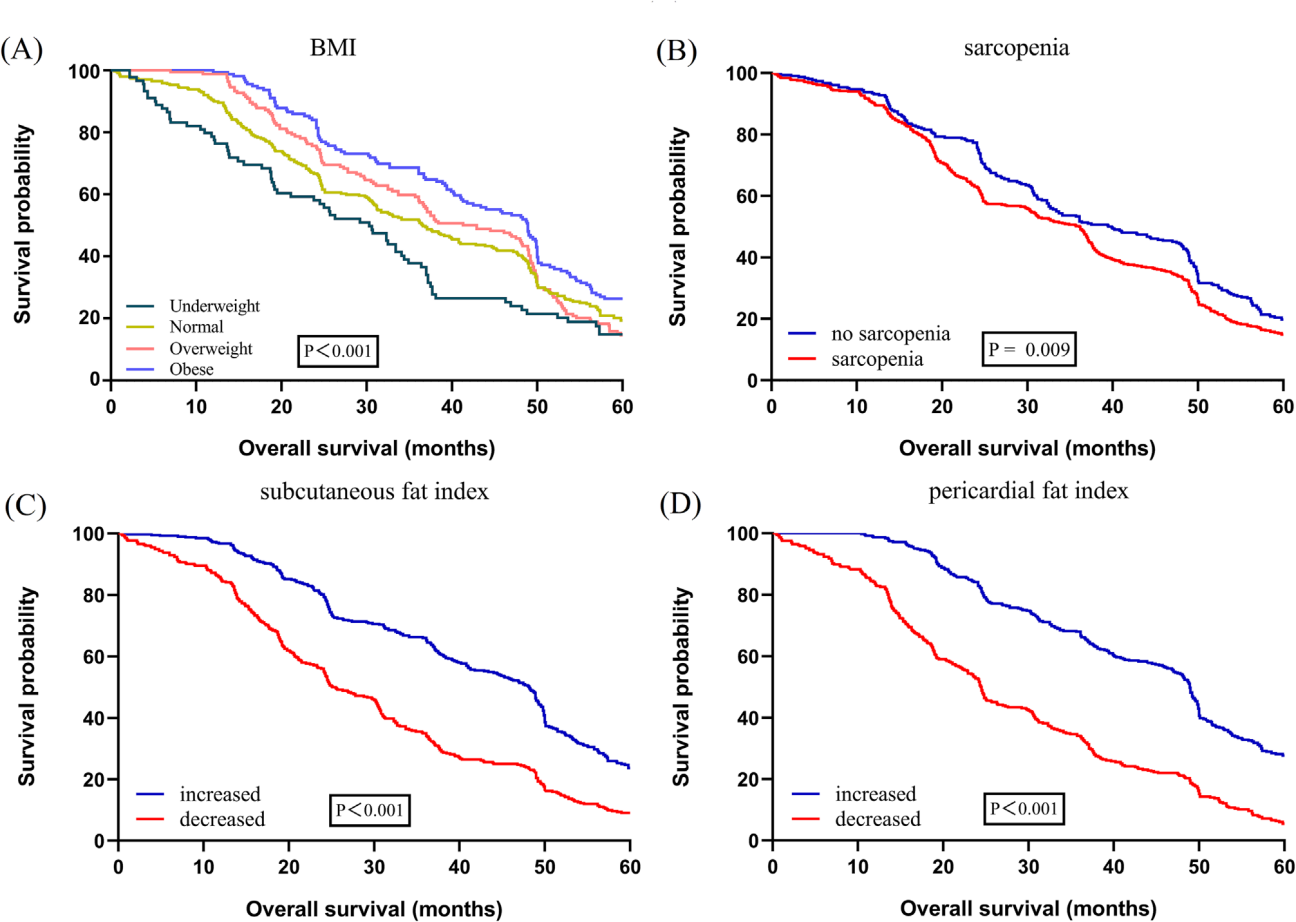
Kaplan–Meier curves show overall survival rate in patients according to body mass index (BMI) category (A), sarcopenia status (B), subcutaneous fat index status (C), and pericardial fat index status (D).

The patients' median survival time was 36.3 months (interquartile range, 19.4–52.4 months). In the Kaplan–Meier analysis, patients with lower BMI (*P* < 0.001), sarcopenia (*P* = 0.009), decreased SFI (*P* < 0.001) and decreased PFI (*P* < 0.001) had lower 5‐year OS rate (*Figure* 4).

In the univariate Cox regression analysis, older age, increased CEA level, coronary calcification, emphysema, higher TNM staging, higher clinical stage, lower BMI and sarcopenia were associated with lower 5‐OS rate. In contrast, patients with higher BMI and increased SFI and PFI had better 5‐year OS rates (*Table* 3).

**Table 3:** Univariable and multivariable analyses of clinical and body composition parameters for 5‐year OS

Characteristic	Univariant analysis		Multivariant analysis	
	HR (95% CI)	*P*	HR (95% CI)	*P*
Age (year)	1.02 (1.01–1.03)	<0.001	1.02 (1.01–1.03)	0.001
Gender	0.94 (0.80–1.11)	0.46		
Smoking history	1.04 (0.93–1.16)	0.51		
Family history	1.10 (0.84–1.46)	0.48		
CEA: increased^a^	1.44 (1.23–1.69)	<0.001	1.00 (0.83–1.18)	0.08
BMI (kg/m^2^) category^b^				
Underweight (<18.5)	1.96 (1.53–2.51)	<0.001	1.62 (1.25–2.10)	<0.001
Normal (18.5–22.9)	Reference		Reference	
Overweight (23.0–24.9)	0.87 (0.72–1.07)	0.21	0.98 (0.80–1.21)	0.85
Obese (≥25)	0.65 (0.53–0.81)	<0.001	0.80 (0.65–1.01)	0.04
Histologic type	0.99 (0.88–1.13)	0.94		
Coronary calcification^c^	1.38 (1.17–1.62)	<0.001	1.04 (0.87–1.24)	0.66
Diabetes mellitus	1.37 (1.18–1.52)	0.12		
Hypertension	1.13 (0.93–1.37)	0.21		
Emphysema^d^	1.36 (1.16–1.59)	<0.001	1.03 (0.87–1.23)	0.67
T	1.16 (1.08–1.25)	<0.001		
1			Reference	
2			1.08 (0.68–1.10)	0.20
3			1.09 (0.63–1.11)	0.21
4			1.19 (0.72–1.28)	0.67
N	1.21 (1.13–1.29)	<0.001		
0			Reference	
1			1.17 (0.87–1.57)	0.11
2			1.21 (0.94–1.73)	0.3
3			1.26 (0.95–1.67)	0.11
M	1.83 (1.56–2.14)	<0.001		
0			Reference	
1			1.50 (0.79–2.25)	0.78
Stage	1.35 (1.25–1.46)	<0.001		
1			Reference	
2			1.05 (0.72–1.53)	0.78
3			1.11 (0.75–1.64)	0.62
4			1.91 (1.05–2.78)	0.94
Surgery^e^	0.54 (0.45–0.64)	<0.001	0.75 (0.60–0.96)	0.02
Sarcopenia	1.42 (1.23–1.52)	<0.001	0.85 (0.72–1.01)	0.08
SFI increased	0.51 (0.43–0.59)	<0.001	0.56 (0.47–0.66)	<0.001
PFI increased	0.41 (0.34–0.48)	<0.001	0.47 (0.40–0.56)	<0.001

Numbers in parentheses are 95% CI. *P* < 0.1 was used for the univariable analysis, and *P* < 0.05 was used for the multivariable analysis. The cutoff values for sarcopenia, increase of SFI and PFI were respectively 14.7 cm^2^/m^2^, 28.9 cm^2^/m^2^ and 85.3 cm^3^/m^2^ for female, and 18.1 cm^2^/m^2^, 21.0 cm^2^/m^2^ and 81 cm^3^/m^2^ for male. Multivariant analysis model was adjusted for the following covariates: age (continuous per year), CEA status (normal/increased), BMI, coronary calcification (no/yes), emphysema (no/yes), T‐N‐M stage, pathologic stage (I/II/III/IV), surgery (no/yes), presence of sarcopenia, SFI status (low/increased) and PFI status (low/increased).

BMI, body mass index; CI, confidence interval; HR, hazard ratio; SFI, subcutaneous fat index; PFI, pericardial fat index.

^a^ The HR was compared with the HR for normal CEA status.

^b^ The HR was compared with the HR for a normal BMI.

^c^ The HR was compared with the HR for no coronary calcification.

^d^ The HR was compared with the HR for no emphysema.

^e^ The HR was compared with the HR for no surgery.

In multivariable Cox regression analysis, after adjusting for CEA, coronary calcification, emphysema, TNM staging, clinical stage, sarcopenia, SFI and PFI, older age (HR = 1.02, 95% CI: 1.01–1.03, *P* = 0.001) and underweight (HR = 1.62, 95% CI: 1.25–2.10, *P* < 0.001) were associated with poorer prognosis. Obesity (HR = 0.80, 95% CI: 0.65–1.0, *P* = 0.04), increased SFI (HR = 0.56, 95% CI: 0.47–0.66, *P* < 0.001) and PFI (HR = 0.47, 95% CI: 0.40–0.56, *P* < 0.001) were associated with better prognosis. No association was noted between sarcopenia and OS (*P* = 0.08).

In subgroup analysis, for patients with stage I–III disease, increased SFI (HR = 0.62, 95% CI: 0.48–0.76, *P* < 0.001) and PFI (HR = 0.43, 95% CI: 0.34–0.54, *P* < 0.001) were associated with better 5‐year OS rate (*Table* S1). Similar results were recorded for patients with stage IV (*Table* S2). For patients whose initial treatment was surgery, the prognostic value of increased SFI (HR = 0.60, 95% CI: 0.44–0.80, *P* = 0.001) and PFI (HR = 0.51, 95% CI: 0.38–0.68, *P* < 0.001) remained and predicted favourable OS (*Table* S3). Non‐surgical patients showed similar results as surgical patients (*Table* S4). No association was noted between sarcopenia and OS in any model (*P* = 0.21, 0.06, 0.30 and 0.07, respectively).

## Discussion

Our study demonstrated that underweight, obesity, PFI and SFI were independent indicators for predicting prognosis of NSCLC patients. Obesity, increased PFI and SFI were associated with better 5‐year OS rates, whereas being underweight was adversely associated with the prognosis. No association was noted between sarcopenia and OS.

Obesity has been proved to be associated with the development of many chronic disease such as cardiovascular disease, type 2 diabetes, non‐alcoholic fatty liver disease, nephropathy and retinopathy.^21^, ^22^, ^23^ In contrast, obese patients have also been reported to show better clinical prognosis in various cancers than their underweight counterparts.^24^, ^25^, ^26^ This phenomenon is known as the obesity paradox. Analogously, the paradox of lung cancer is that high BMI increases the incidence of cancer. However, in a large cohort of 54 631 lung resection NSCLC patients, obese patients were found to have improved survival compared with normal weight and underweight patients.^27^ Liu et al. also found that obesity is related to longer progression‐free survival in lung cancer patients treated with immune checkpoint inhibitors.^28^ However, because BMI cannot differentiate between adipose tissue and skeletal muscle or delineate adipose tissue distribution, the reason for the complicated relationship between obesity and prognosis in lung cancer patients remains unclear.

Previous studies have shown that cancer cachexia is characterized by a decrease in adipose tissue and skeletal muscle mass, leading to grievous weight loss. Approximately 50% of cancer patients suffer from cachexia, which directly causes at least 20% of cancer‐related deaths.^29^ Obesity can compensate for fat loss in cancer patients, aggregate adipose tissue as a protective tool against cancer progression and improve the response to treatment.^30^ Adipose tissues mainly include subcutaneous fat tissue and visceral fat. Lee et al. found that greater volume of subcutaneous adipose tissue has a favourable effect on progression‐free survival in NSCLC patients.^31^ Additional studies have evaluated the association between muscle‐derived variables, BMI, obesity and OS and found a trend toward improvement in visceral obesity and OS.^11^ Previous studies have shown that pericardial fat and visceral fat originate from the same source and that pericardial fat is significantly correlated with visceral fat.^32^ In our study, we measured the amount of skeletal muscle area, subcutaneous fat area and pericardial fat volume and generated cut‐off values for the definition of sarcopenia and decreased SFI and PFI according to sex. After adjusting for clinical and body composition variables, we demonstrated that obesity and increased PFI and SFI were independently associated with an improved 5‐year OS rate. In comparison, sarcopenia was not significantly associated with OS. This may be because cancer patients generally require more energy, and fat is an important source of energy. Fat depletion precedes, or even occurs without, muscle wasting in cancer patients.^33^, ^34^ This pattern has also been observed in gastrointestinal tract cancer patients.^35^ Fat depletion is characterized by enhanced lipolysis and reduced adipocyte size.^36^ The decrease in energy reserves and disruption of the energy balance caused by fat deficiency may be closely related to the shortened survival time of cancer patients.^37^ Thus, we attribute the obesity paradox in NSCLC patients to adipose tissue rather than skeletal muscle.

In our subgroup analysis, the 5‐year OS rate in stage I–III NSCLC patients was associated with increased PFI and SFI but not sarcopenia. These results were in accordance with those of a previous report on adipopexia, instead of sarcopenia, using preoperative PET/CT, in which the 5‐year OS was reduced.^10^ We found similar results in stage IV NSCLC patients; increased PFI and SFI were associated with better OS. Interestingly, we found that increased SFI and PFI were independent risk factors for predicting prognosis in NSCLC patients, regardless of whether they were initially treated surgically or non‐surgically, whereas sarcopenia was not. Skeletal muscle depletion was associated with prognosis in NSCLC, which was related to the shrinkage of morphometric mass, whereas the single assessment of pectoralis muscle mass was insufficient to predict both short‐ and long‐term outcomes.^17^ Increased PFI and SFI may be markers of physiological reserves with respect to OS, with higher PFI and SFI indicating better reserves. In addition, increased adiponectin and leptin production in visceral adipose tissue may be associated with improved OS in lung cancer.^38^ Hence, for newly diagnosed NSCLC patients, the identification of at‐risk patients will allow for accurate risk stratification and treatment decisions, as well as the provision of appropriate supportive care. Although it may not be the best time to intervene during NSCLC diagnosis, in clinical practice, it is critical to raise awareness among physicians and patients regarding treatment strategies. Furthermore, the early identification of patients at risk will improve interventions targeting fat loss.

Our study has several limitations. First, this was a retrospective and single‐centre study, which might have resulted in a patient selection bias. Thus, prospective multicentre investigations are required to validate our results. Second, skeletal muscle area and subcutaneous fat area were measured at the Th4 level instead of L3, because most of our patients did not undergo abdominal scanning. Although L3 is considered the standard level for body composition measurement, Th4 was confirmed to be an eligible alternative with better clinical feasibility. Finally, because the reference values for SMI and SFI at the Th4 level and PFI have not been established, we used X‐tile to generate cut‐off values. Further validation is needed for larger populations and other races.

In conclusion, our study showed that increased PFI and SFI were associated with a higher 5‐year OS rate, independent of sarcopenia, in NSCLC patients. PFI and SFI are potential early survival markers that can be obtained from baseline chest CT scans for survival stratification and may help identify patients at risk of non‐cancer‐related death and cancer cachexia, thereby providing targeted nutritional support to high‐risk patients.

## Conflict of interest

We have no subject overslap with previously published works in this study. I would like to declare on behalf that the work described was original research that has not been published previously, and not under consideration for publication elsewhere, in whole or in part. No conflict of interest exists in the submission of this manuscript, and the manuscript is approved by all authors for publication. All the authors listed have approved the manuscript that is enclosed. All the authors certify that they comply with the ethical guidelines for publishing in the Journal of Journal of Cachexia, Sarcopenia and Muscle. This study has been approved by the Institutional Review Committee.

## Supporting information


**Table S1.** Univariable and multivariable analyses of clinical and body composition parameters in 5‐year OS for stage I‐III NSCLC patients.

**Table S2.** Univariable and multivariable analyses of clinical and body composition parameters in 5‐year OS for stage IV NSCLC patients.

**Table S3.** Univariable and multivariable analyses of clinical and body composition parameters in 5‐year OS for surgical patients.

**Table S4.** Univariable and multivariable analyses of clinical and body composition parameters in 5‐year OS for non‐surgical patients.
