# Supplementary material for: Increased adipose tissue is associated with improved overall survival, independent of skeletal muscle mass in non‐small cell lung cancer
Source: J Cachexia Sarcopenia Muscle. 2023 Sep 19;14(6):2591–601. doi: 10.1002/jcsm.13333 (PMC10751412; doi:10.1002/jcsm.13333)
Supplement: Supplementary file 1 — Table S1. Univariable and multivariable analyses of clinical and body composition parameters in 5‐year OS for stage I‐III NSCLC patients. [file JCSM-14-2591-s004.docx]

**Table S1** Univariable and multivariable analyses of clinical and body composition parameters in 5-year OS for stage I-III NSCLC patients

|  | Univariant analysis | |  | Multivariant analysis | |
| --- | --- | --- | --- | --- | --- |
| Characteristic | HR (95% CI) | *P* |  | HR (95% CI) | *P* |
| Age (year) | 1.01 (1.00-1.03) | 0.03 |  | 1.01 (0.99-1.03) | 0.08 |
| Gender | 0.92 (0.72-1.12) | 0.53 |  |  |  |
| Smoking history | 1.09 (0.85-1.39) | 0.52 |  |  |  |
| Family history | 1.06 (0.84-1.45) | 0.86 |  |  |  |
| CEA: increased^a^ | 1.31 (1.01-1.68) | 0.04 |  | 1.18 (0.95-1.38) | 0.88 |
| BMI (kg/m^2^) category^b^ |  |  |  |  |  |
| Underweight (<18.5) | 1.54 (1.01-2.36) | 0.04 |  | 1.11 (0.71-1.72) | 0.06 |
| Normal (18.5-22.9) | reference |  |  | reference |  |
| Overweight (23.0-24.9) | 0.92 (0.70-1.21) | 0.54 |  | 0.85 (0.64-1.12) | 0.26 |
| Obese (≥25) | 0.60(0.45-0.82) | 0.001 |  | 0.71 (0.52-0.96) | 0.03 |
| Histologic type | 0.69 (0.37-1.27) | 0.24 |  |  |  |
| Coronary calcification^c^ | 1.57 (1.28-1.97) | <0.001 |  | 1.21 (0.93-1.55) | 0.15 |
| Diabetes mellitus | 1.07 (0.77-1.64) | 0.89 |  |  |  |
| Hypertension | 1.06 (0.81-1.41) | 0.66 |  |  |  |
| Emphysema^d^ | 1.45 (1.16-1.82) | 0.001 |  | 1.25 (0.98-1.59) | 0.07 |
| Surgery^e^ | 0.65 (0.52-0.81) | <0.001 |  | 0.68 (0.53-0.87) | 0.02 |
| Sarcopenia | 1.26 (1.09-1.68) | 0.21 |  |  |  |
| SFI increased | 0.55 (0.44-0.69) | <0.001 |  | 0.62 (0.48-0.76) | <0.001 |
| PFI increased | 0.41 (0.32-0.51) | <0.001 |  | 0.43 (0.34-0.54) | <0.001 |

* Numbers in parentheses are 95% CI. *P* < 0.1 was used for the univariable analysis, and *P* < 0.05 was used for the multivariable analysis. BMI, body mass index; CI, confidence interval; HR, hazard ratio; SFI, subcutaneous fat index; PFI, pericardial fat index. The cutoff values for sarcopenia, increase of SFI and PFI were respectively 14.7 cm^2^/m^2^, 28.9 cm^2^/m^2^ and 85.3 cm^3^/m^2^ for female, and 18.1 cm^2^/m^2^, 21.0 cm^2^/m^2^ and 81cm^3^/m^2^ for male. Multivariant analysis model was adjusted for the following covariates: age (continuous per year), CEA status (normal/increased), BMI, coronary calcification (no/yes), emphysema (no/yes), surgery (no/yes), SFI status (low/increased) and PFI status (low/increased).

^a^The HR was compared with the HR for normal CEA status.

^b^The HR was compared with the HR for a normal BMI.

^c^The HR was compared with the HR for no coronary calcification.

^d^The HR was compared with the HR for no emphysema.

^e^The HR was compared with the HR for no surgery.
