# Supplementary material for: Increased adipose tissue is associated with improved overall survival, independent of skeletal muscle mass in non‐small cell lung cancer
Source: J Cachexia Sarcopenia Muscle. 2023 Sep 19;14(6):2591–601. doi: 10.1002/jcsm.13333 (PMC10751412; doi:10.1002/jcsm.13333)
Supplement: Supplementary file 2 — Table S2. Univariable and multivariable analyses of clinical and body composition parameters in 5‐year OS for stage IV NSCLC patients. [file JCSM-14-2591-s003.docx]

**Table S2** Univariable and multivariable analyses of clinical and body composition parameters in 5-year OS for stage IV NSCLC patients

|  | Univariant analysis | |  | Multivariant analysis | |
| --- | --- | --- | --- | --- | --- |
| Characteristic | HR (95% CI) | *P* |  | HR (95% CI) | *P* |
| Age (year) | 1.01 (1.00-1.03) | 0.02 |  | 1.02 (1.00-1.03) | 0.03 |
| Gender | 1.03 (0.82-1.29) | 0.81 |  |  |  |
| Smoking history | 1.08 (0.86-1.35) | 0.85 |  | ... | ... |
| Family history | 1.22 (0.84-1.78) | 0.30 |  | ... | ... |
| CEA: increased^a^ | 1.07 (0.85-1.34) | 0.58 |  | ... | ... |
| BMI (kg/m^2^) category^b^ |  |  |  |  |  |
| Underweight (<18.5) | 1.97 (1.45-2.69) | <0.001 |  | 2.16 (1.52-3.00) | <0.001 |
| Normal (18.5-22.9) | reference |  |  | reference |  |
| Overweight (23.0-24.9) | 0.90 (0.67-1.22) | 0.50 |  | 0.93 (0.63-1.34) | 0.45 |
| Obese (≥25) | 0.74 (0.54-1.01) | 0.06 |  | 0.86 (0.69-1.22) | 0.78 |
| Histologic type | 1.10 (0.85-1.43) | 0.48 |  | ... |  |
| Coronary calcification^c^ | 1.26 (1.00-1.58) | 0.05 |  | 1.06 (0.77-1.30) | 0.76 |
| Diabetes mellitus | 1.65 (1.46-1.91) | 0.12 |  |  |  |
| Hypertension | 1.17 (0.90-1.52) | 0.26 |  |  |  |
| Emphysema^d^ | 1.20 (0.96-1.50) | 0.12 |  |  |  |
| Surgery^e^ | 0.69 (0.41-1.13) | 0.14 |  |  |  |
| Sarcopenia | 1.17 (1.01-1.36) | 0.04 |  | 0.73 (0.57-0.92) | 0.06 |
| SFI increased | 0.54 (0.43-0.68) | <0.001 |  | 0.50 (0.39-0.64) | <0.001 |
| PFI increased | 0.46 (0.37-0.58) | <0.001 |  | 0.53 (0.41-0.68) | <0.001 |

* Numbers in parentheses are 95% CI. *P* < 0.1 was used for the univariable analysis, and *P* < 0.05 was used for the multivariable analysis. BMI, body mass index; CI, confidence interval; HR, hazard ratio; SFI, subcutaneous fat index; PFI, pericardial fat index. The cutoff values for sarcopenia, increase of SFI and PFI were respectively 14.7 cm^2^/m^2^,28.9 cm^2^/m^2^ and 85.3 cm^3^/m^2^ for female, and 18.1 cm^2^/m^2^, 21.0 cm^2^/m^2^ and 81 cm^3^/m^2^ for male. Multivariant analysis model was adjusted for the following covariates: age (continuous per year), BMI, coronary calcification (no/yes), presence of sarcopenia, SFI status (low/increased) and PFI status (low/increased).

^a^The HR was compared with the HR for normal CEA status.

^b^The HR was compared with the HR for a normal BMI.

^c^The HR was compared with the HR for no coronary calcification.

^d^The HR was compared with the HR for no emphysema.

^e^The HR was compared with the HR for no surgery.
