# Supplementary material for: Increased adipose tissue is associated with improved overall survival, independent of skeletal muscle mass in non‐small cell lung cancer
Source: J Cachexia Sarcopenia Muscle. 2023 Sep 19;14(6):2591–601. doi: 10.1002/jcsm.13333 (PMC10751412; doi:10.1002/jcsm.13333)
Supplement: Supplementary file 3 — Table S3. Univariable and multivariable analyses of clinical and body composition parameters in 5‐year OS for surgical patients. [file JCSM-14-2591-s002.docx]

**Table S3** Univariable and multivariable analyses of clinical and body composition parameters in 5-year OS for surgical patients

|  | Univariant analysis | |  | Multivariant analysis | |
| --- | --- | --- | --- | --- | --- |
| Characteristic | HR (95% CI) | *P* |  | HR (95% CI) | *P* |
| Age (year) | 1.01 (0.99-1.02) | 0.63 |  |  |  |
| Gender | 1.14 (0.85-1.53) | 0.37 |  |  |  |
| Smoking history | 1.17 (0.78-1.78) | 0.44 |  |  |  |
| Family history | 1.21 (0.74-1.96) | 0.45 |  |  |  |
| CEA: increased^a^ | 1.72 (1.24-2.38) | 0.001 |  | 1.74 (1.22-2.47) | 0.02 |
| BMI (kg/m^2^) category^b^ |  |  |  |  |  |
| Underweight (<18.5) | 1.35 (0.68-2.70) | 0.39 |  | 1.41 (0.70-2.89) | 0.33 |
| Normal (18.5-22.9) | reference |  |  | reference |  |
| Overweight (23.0-24.9) | 0.94 (0.65-1.36) | 0.80 |  | 0.89 (0.63-1.25) | 0.48 |
| Obese (≥25) | 0.65 (0.45-0.95) | 0.03 |  | 0.73 (0.50-1.09) | 0.12 |
| Histologic type | 0.86 (0.40-1.85) | 0.71 |  |  |  |
| Coronary calcification^c^ | 1.55 (1.15-2.08) | 0.004 |  | 1.36 (0.98-1.89) | 0.07 |
| Diabetes mellitus | 1.06 (0.72-1.51) | 0.55 |  |  |  |
| Hypertension | 1.10 (0.77-1.57) | 0.60 |  |  |  |
| Emphysema^d^ | 1.55 (1.16-2.07) | 0.003 |  | 1.58 (1.14-2.20) | 0.06 |
| stage |  |  |  |  |  |
| 1 | reference |  |  | reference |  |
| 2 | 1.26 (0.89-1.77) | 0.19 |  | 1.02 (0.70-1.47) | 0.94 |
| 3 | 1.28 (0.89-1.86) | 0.20 |  | 1.14 (0.78-1.68) | 0.50 |
| 4 | 1.98(1.15-3.41) | 0.01 |  | 1.62 (0.93-2.82) | 0.09 |
| Sarcopenia | 1.26 (0.95-1.45) | 0.30 |  |  |  |
| SFI increased | 0.61 (0.46-0.81) | 0.001 |  | 0.60 (0.44-0.80) | 0.001 |
| PFI increased | 0.50 (0.38-0.67) | <0.001 |  | 0.51 (0.38-0.68) | <0.001 |

* Numbers in parentheses are 95% CI. *P* < 0.1 was used for the univariable analysis, and *P* < 0.05 was used for the multivariable analysis. BMI, body mass index; CI, confidence interval; HR, hazard ratio; SFI, subcutaneous fat index; PFI, pericardial fat index. The cutoff values for sarcopenia, increase of SFI and PFI were respectively 14.7 cm^2^/m^2^,28.9 cm^2^/m^2^ and 85.3 cm^3^/m^2^ for female, and 18.1 cm^2^/m^2^, 21.0 cm^2^/m^2^ and 81 cm^3^/m^2^ for male. Multivariant analysis model was adjusted for the following covariates: CEA status (normal/increased), BMI, coronary calcification (no/yes), emphysema (no/yes), pathologic stage (I/II/III/IV), SFI status (low/increased) and PFI status (low/increased).

^a^The HR was compared with the HR for normal CEA status.

^b^The HR was compared with the HR for a normal BMI.

^c^The HR was compared with the HR for no coronary calcification.

^d^The HR was compared with the HR for no emphysema.
