# Supplementary material for: Increased adipose tissue is associated with improved overall survival, independent of skeletal muscle mass in non‐small cell lung cancer
Source: J Cachexia Sarcopenia Muscle. 2023 Sep 19;14(6):2591–601. doi: 10.1002/jcsm.13333 (PMC10751412; doi:10.1002/jcsm.13333)
Supplement: Supplementary file 4 — Table S4. Univariable and multivariable analyses of clinical and body composition parameters in 5‐year OS for non‐surgical patients. [file JCSM-14-2591-s001.docx]

**Table S4** Univariable and multivariable analyses of clinical and body composition parameters in 5-year OS for non-surgical patients

|  | Univariant analysis | |  | Multivariant analysis | |
| --- | --- | --- | --- | --- | --- |
| Characteristic | HR (95% CI) | *P* |  | HR (95% CI) | *P* |
| Age (year) | 1.01 (1.00-1.02) | 0.01 |  | 1.02 (1.01-1.03) | <0.001 |
| Gender | 1.04 (0.85-1.27) | 0.69 |  |  |  |
| Smoking history | 1.03 (0.79-1.33) | 0.85 |  |  |  |
| Family history | 1.07 (0.76-1.50) | 0.70 |  |  |  |
| CEA: increased^a^ | 1.07 (0.89-1.30) | 0.46 |  |  |  |
| BMI (kg/m^2^) category^b^ |  |  |  |  |  |
| Underweight (<18.5) | 1.83 (1.40-2.40) | <0.001 |  | 1.69 (1.27-2.24) | <0.001 |
| Normal (18.5-22.9) | reference |  |  | reference |  |
| Overweight (23.0-24.9) | 0.88 (0.68-1.14) | 0.33 |  | 0.95 (0.60-1.16) | 0.74 |
| Obese (≥25) | 0.71(0.54-0.92) | 0.01 |  | 0.88 (0.67-1.16) | 0.38 |
| Histologic type | 0.89 (0.61-1.23) | 0.54 |  |  |  |
| Coronary calcification^c^ | 1.26 (1.04-1.53) | 0.02 |  | 0.95 (0.76-1.18) | 0.65 |
| Diabetes mellitus | 1.15 (0.96-1.42) | 0.46 |  |  |  |
| Hypertension | 1.09 (0.87-1.38) | 0.44 |  |  |  |
| Emphysema^d^ | 1.16 (0.96-1.40) | 0.14 |  |  |  |
| stage |  |  |  |  |  |
| 1 | reference |  |  | reference |  |
| 2 | 1.18 (0.55-2.55) | 0.67 |  | 1.02 (0.47-2.24) | 0.95 |
| 3 | 1.65 (0.93-2.94) | 0.08 |  | 1.70 (0.94-3.07) | 0.07 |
| 4 | 1.99 (1.14-3.47) | 0.02 |  | 1.82 (1.03-3.22) | 0.04 |
| Sarcopenia | 1.19 (0.95-1.36) | 0.05 |  | 0.81 (0.66-0.98) | 0.07 |
| SFI increased | 0.50 (0.41-0.61) | <0.001 |  | 0.53 (0.43-0.64) | <0.001 |
| PFI increased | 0.40 (0.33-0.49) | <0.001 |  | 0.47 (0.38-0.68) | <0.001 |

* Numbers in parentheses are 95% CI. *P* < 0.1 was used for the univariable analysis, and *P* < 0.05 was used for the multivariable analysis. BMI, body mass index; CI, confidence interval; HR, hazard ratio; SFI, subcutaneous fat index; PFI, pericardial fat index. The cutoff values for sarcopenia, increase of SFI and PFI were respectively 14.7 cm^2^/m^2^,28.9 cm^2^/m^2^ and 85.3 cm^3^/m^2^ for female, and 18.1 cm^2^/m^2^, 21.0cm^2^/m^2^ and 81cm^3^/m^2^ for male. Multivariant analysis model was adjusted for the following covariates: age (continuous per year), BMI, coronary calcification (no/yes), pathologic stage (I/II/III/IV), presence of sarcopenia, SFI status (low/increased) and PFI status (low/increased).

^a^The HR was compared with the HR for normal CEA status.

^b^The HR was compared with the HR for a normal BMI.

^c^The HR was compared with the HR for no coronary calcification.

^d^The HR was compared with the HR for no emphysema.
